# Transcription factors: key regulatory targets of vascular smooth muscle cell in atherosclerosis

**DOI:** 10.1186/s10020-022-00586-2

**Published:** 2023-01-05

**Authors:** Yu Jiang, Hai-Yan Qian

**Affiliations:** Center for Coronary Heart Disease, Department of Cardiology, Fu Wai Hospital, National Center for Cardiovascular Diseases of China, State Key Laboratory of Cardiovascular Disease, Chinese Academy of Medical Sciences and Peking Union Medical College, 167 Beilishi Rd, Beijing, 100037 China

**Keywords:** Transcription factors, Vascular smooth muscle cells, Atherosclerosis

## Abstract

Atherosclerosis (AS), leading to gradual occlusion of the arterial lumen, refers to the accumulation of lipids and inflammatory debris in the arterial wall. Despite therapeutic advances over past decades including intervention or surgery, atherosclerosis is still the most common cause of cardiovascular diseases and the main mechanism of death and disability worldwide. Vascular smooth muscle cells (VSMCs) play an imperative role in the occurrence of atherosclerosis and throughout the whole stages. In the past, there was a lack of comprehensive understanding of VSMCs, but the development of identification technology, including in vivo single-cell sequencing technology and lineage tracing with the CreERT2-loxP system, suggests that VSMCs have remarkable plasticity and reevaluates well-established concepts about the contribution of VSMCs. Transcription factors, a kind of protein molecule that specifically recognizes and binds DNA upstream promoter regions or distal enhancer DNA elements, play a key role in the transcription initiation of the coding genes and are necessary for RNA polymerase to bind gene promoters. In this review, we highlight that, except for environmental factors, VSMC genes are transcriptionally regulated through complex interactions of multiple conserved cis-regulatory elements and transcription factors. In addition, through a series of transcription-related regulatory processes, VSMCs could undergo phenotypic transformation, proliferation, migration, calcification and apoptosis. Finally, enhancing or inhibiting transcription factors can regulate the development of atherosclerotic lesions, and the downstream molecular mechanism of transcriptional regulation has also been widely studied.

## Introduction

Atherosclerosis (AS) is a chronic inflammatory process driven by cholesterol accumulation coupled to inflammatory and fibrotic reactions within the arterial wall, and it remains the leading cause of death worldwide (Libby et al. 2011; Tabas et al. 2015). During the development of atherosclerosis, low-density lipoprotein (LDL) particles enter the tunica intima, where they get oxidized and convert to oxidized low-density lipoprotein (oxLDL) (Goikuria et al. 2018). Macrophages, in turn, scavenge oxLDL and convert into foam cells, then foam cells gradually build up fatty streaks and produce cytokines, ultimately leading to the migration and proliferation of medial vascular smooth muscle cells (VSMCs) and the emergence of unstable plaques (Owens et al. 2004). The acute rupture of these unstable plaques causes local thrombus formation, resulting in partial stenosis or total occlusion of the vascular lumen and severe cardiovascular or cerebrovascular events such as myocardial infarction and stroke (Bentzon et al. 2014). Despite the numerous progress in atherosclerotic research during the recent several decades, the definitive evidence of the origin and function of many cells involved in atherosclerosis, as well as the downstream molecular mechanisms of how they accelerate lipoprotein oxidation, inflammation and immunity, is still unclear (Bentzon et al. 2014; Libby et al. 2011). Understanding these mechanisms involved in atherosclerosis and plaque destabilization is crucial for developing new therapeutic strategies (Rader and Daugherty 2008).

In normal vessels, VSMCs are quiescent within the medial layer, where they are responsible for arterial contraction, blood distribution and production of extracellular matrix (ECM). The roles of VSMCs are indispensable for maintaining the normal functions of arterial vessels, including elastic recoil in large elastic arteries and modulation of arterial diameter in middle arteries and arterioles (Basatemur et al. 2019). However, when exposed to lipid deposition and inflammatory factors, VSMCs play an important role in the occurrence and development of atherosclerotic plaques and throughout the whole stages. Under these circumstances, VSMCs could undergo phenotypic modulation with higher expression of ECM components and matrix metalloproteinases (MMPs), also increase levels of secretory organelles and release pro-inflammatory cytokines (Clarke et al. 2008). Besides, activation of VSMC proliferation and migration has also been proved in atherosclerosis (Allahverdian et al. 2018; Bennett et al. 2016; Wirka et al. 2019).

Transcription factors have been proven to modulate the process of development and differentiation by influencing RNA polymerase in eukaryotic cells (Vaquerizas et al. 2009), most transcription factors have two distinct structural domains including a sequence-specific DNA-binding domain and a transcription activation or repression domain that interacts with various cofactors. Both of which are indispensable for the regulatory function of transcription factors. By targeting binding multiple conserved cis-regulatory elements, transcription factors can participate in regulating gene expression (Papavassiliou and Papavassiliou 2016). However, given the complexity and vulnerability of the transcriptional regulatory network, regulating transcription is not an absolutely stable process, which is easily disturbed during pathological progression.

The development of atherosclerotic lesions is regulated by many factors, among which the noteworthy regulation of environmental factors on VSMCs has been widely confirmed (Majesky 2007; Owens et al. 2004; Raines and Ferri 2005). Besides, tremendous progress has been made in the last decade to identify the molecular mechanism of transcriptional regulators in VSMCs. Compared with cell-specific alternative splicing and posttranslational modifications, activation or inhibition of transcription factors in transcription level has been considered more significant to regulate rates and patterns of gene expression in response to injury. VSMC genes are transcriptionally regulated through complex interactions of transcription factors and conserved cis-regulatory elements (Alexander and Owens 2012). Moreover, the expression of VSMC-selective genes is not dependent on the regulation of individual genes but depends on interactions of multiple gene expression (Zhang et al. 2002). VSMCs can undergo a series of pathological and physiological changes in transcription related regulatory processes. The downstream molecular mechanism of transcriptional regulation has also been widely studied.

**Fig. 1:**
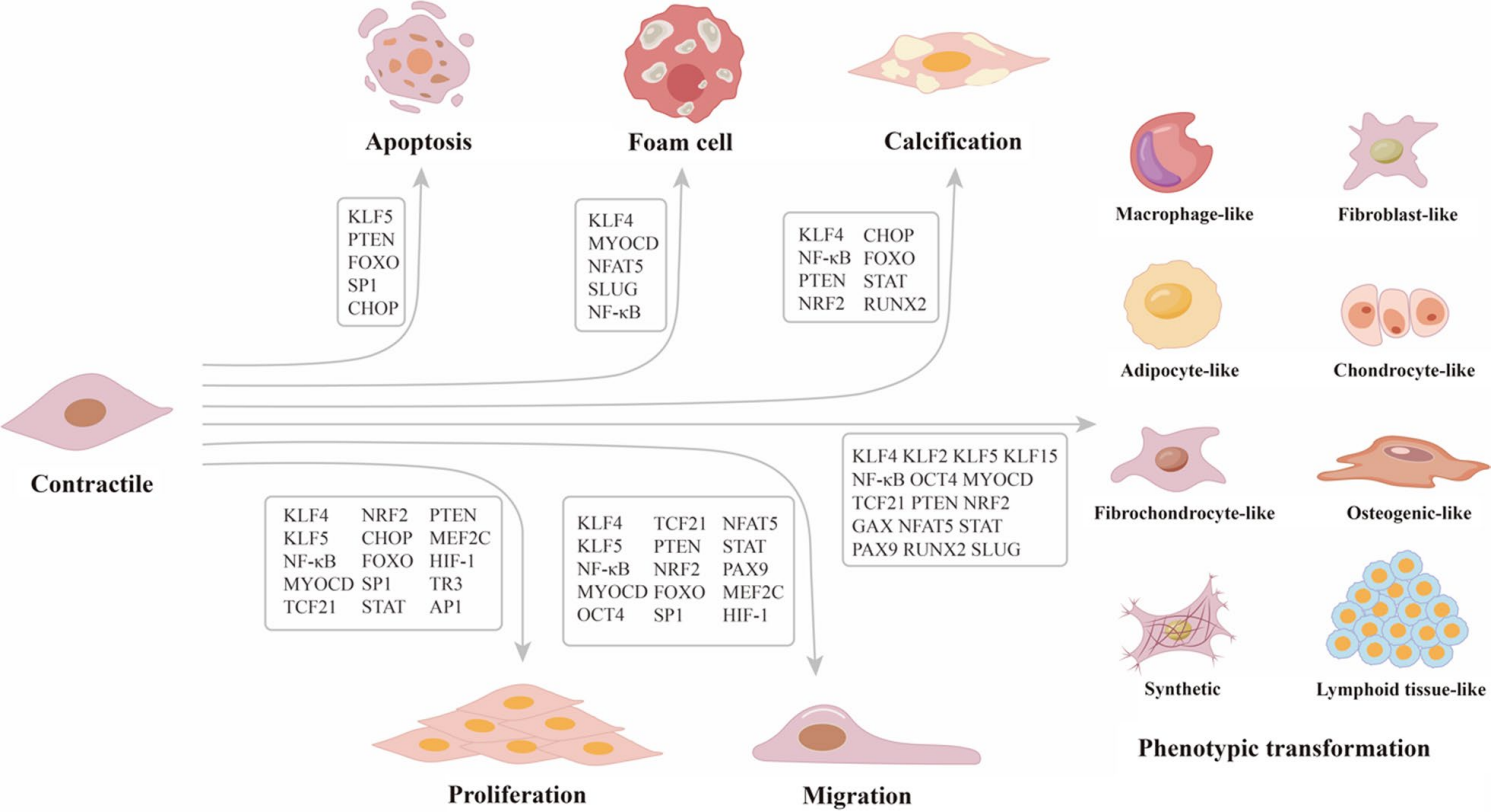
Schematic of transcription factors involved in VSMC regulation. Through transcription-related regulatory processes, VSMCs could undergo phenotypic transformation and a series of atherosclerosis-related pathological processes. For the specific mechanisms of transcription factors during the atherosclerotic lesions, please refer to the main text

This review summarizes the effects of different transcription factors on VSMCs during the atherosclerotic process (Table 1), including phenotypic transformation, foam cell formation, proliferation, migration, calcification and apoptosis (Fig. 1). In addition, enhancing or inhibiting transcription factors could regulate the development of atherosclerotic lesions, which provides a new direction and understanding of atherosclerotic treatment.

**Table 1:** VSMC regulation by Transcription factor in response to atherosclerosis

TFs	Regulation	Targets	Effects on VSMC
KLF4	↑	α-SMA, SM22α, TAGLN, MYH11, LGALS3, OCT4, MYOCD, P21, Col 8A1, RUNX2	Phenotypic transformation, foam cell, proliferation, migration, calcification
KLF2	↑	MYH11, OCT4	Phenotypic transformation
KLF5	↑	MYH11, OCT4, miR-29a, BCL-2	Phenotypic transformation, proliferation, migration, apoptosis
KLF15	↓	p300	Phenotypic transformation
NF-κB	↑	KLF4, CXCL13, CCL19, VCAM1, ICAM1, LOX-1, cyclin B1, CDK1, IL-6, ANKH, ALP	Phenotypic transformation, foam cell, proliferation, migration, calcification
MYOCD	↓	α-SMA, SM22α, SMMHC, CNN1, SMMLCK, CD68, ABCA1, LOX-1, p65, miR-1	Phenotypic transformation, foam cell, proliferation, migration
OCT4	↑	MYH11, MMP-3, MMP-13	Phenotypic transformation, migration
TCF21	↑	TAGLN, CNN1, ACTA2, SCA1, LGALS3, SRF, MYOCD, MMP-1, MMP-3	Phenotypic transformation, proliferation, migration
PTEN	↓	SRF, PI3K, MCP-1, p70, NF-κB, caspase-3	Phenotypic transformation, proliferation, migration, apoptosis, calcification
NRF2	↓	HO-1, NQO-1	Phenotypic transformation, proliferation, migration, calcification
CHOP	↑	KLF4, BCL-2, BIM, ERO1-α, PiT-1	Proliferation, calcification, apoptosis
FOXO	↑	CYR61, MMP-2, MMP-13, TIMP-1, TIMP-2, TIMP-3, BIM, APAF1, RUNX2	Proliferation, migration, apoptosis, calcification
GAX	↓	Integrinα2	Phenotypic transformation
SP1	↑	KLF4, TRAIL, MMP-9	Proliferation, migration, apoptosis
NFAT5	↑	α-SMA, SOD1, PLIN2, FABP3, PPARD, ABCA1	Phenotypic transformation, foam cell, migration
STAT	↑	cyclin D1, survivin, RUNX2, miR-92a, LOXL1-AS1, MMP-2, ICAM-1, IP-10, RUNX2	Phenotypic transformation, proliferation, migration, calcification
PAX9	↑	α-SMA, SM22α, PCNA, MMP-2, MMP-9	Phenotypic transformation, migration
RUNX2	↑	MYOCD, RANKL, osteocalcin, sclerostin, α-SMA, SM22α, ALP, Col IA1	Phenotypic transformation, calcification
SLUG	↑	COX-2, ABCA1, ABCG1	Phenotypic transformation, foam cell
MEF2C	↓	–	Proliferation, migration
HIF-1	↑	MIF	Proliferation, migration
TR3	↑	p27	Proliferation
AP1	↑	cyclin A	Proliferation

## The impacts of transcription factors on VSMCs

### Phenotypic switch and foam cell formation

#### The transcription factors related to the phenotypic switch

VSMCs expressing high levels of contractile proteins are generally described as contractile phenotype (Poole et al. 1971), whereas, in atherosclerotic plaque, VSMCs undergo the phenotypic switch, also known as de-differentiation, to the proliferative synthetic phenotype. This process is characterized by loss of expression of VSMC specific marker genes, such as ACTA2, MYH11, TAGLN and CNN1 (Owens et al. 2004). In addition, the activation of VSMC proliferation and migration has also been associated with the synthetic phenotype (Lee et al. 1997). Although VSMC proliferation is beneficial in advanced plaques, for example, stabilizing the fibrous cap to prevent acute rupture, more recent studies suggest that inhibiting VSMC phenotypic switch may prevent the progression of atherosclerotic lesions (Huff and Pickering 2015; Vengrenyuk et al. 2015). Numerous transcription factors play different roles in phenotypic regulation.

##### MYOCD, PTEN and TCF21

Myocardin (MYOCD), a coactivator of serum response factor (SRF), is involved in lipid metabolism, vascular inflammation, development of postnatal and embryonic vasculature (Huang et al. 2015; Sward et al. 2016) and the differentiation of cardiomyocyte and VSMC lineages (Wang et al. 2001). MYOCD and SRF complex increases expression of VSMC differentiation markers by binding the CArG box of target promoters to form a MYOCD-SRF-CArG box complex (Chen et al. 2002; Wang et al. 2003), overexpression of MYOCD up-regulates the expression of multiple contractile genes and prevents atherosclerotic lesions. Raphel et al. report that MYOCD overexpression increases the gene markers of contractile phenotype, carbachol-induced contractile function and the development of VSMC-like cells from human embryonic stem cells (Raphel et al. 2012). Wen et al. demonstrate that miR-139-5p could negatively regulate MYOCD expression and down-regulate the expression of contractile VSMC marker genes (Wen et al. 2022). Besides, the miR-145 mimic-transduced VSMCs from atherosclerotic patients show a significant increase in MYOCD expression and further promote phenotypic switch of VSMCs from a proliferative to a contractile state (Zhang et al. 2016). However, the protective effect of this complex could be changed through interaction with other transcription factors. Transcription factor 21 (TCF21), a basic-helix-loop-helix transcription factor that contributes to the risk of coronary heart disease, blocks the MYOCD-SRF pathway via direct TCF21-MYOCD interaction and results in a less differentiated phenotype (Nagao et al. 2020). Besides, Phosphatase and tensin homolog (PTEN) is a dual-specificity protein and lipid phosphatase that suppresses pathological vascular remodeling, including de-differentiation, proliferation and inflammatory cytokine production (Furgeson et al. 2010). PTEN interacts with the N-terminal domain of SRF, which facilitates selective binding of SRF on the MYH11 and ACTA2 promoters. Therefore, PTEN-SRF interaction has an indispensable role in regulating the differentiated phenotype. Decreased expression of PTEN has been observed in human atherosclerotic lesions (Horita et al. 2016). PTEN depletion promotes enhanced Phosphoinositide 3-kinase (PI3K)/protein kinase B (AKT)/mammalian target of rapamycin (mTOR) signaling under basal conditions and induces VSMC phenotypic modulation (Furgeson et al. 2010). Nicotinate-curcumin inhibits AngII-induced VSMC phenotype switching via upregulation of PTEN expression and blocking the activation of phosphorylated AKT (Sun et al. 2021). Besides, decreased expression of PTEN induces upregulation of nuclear factor kappa-B (NF-κB) and NF-κB-dependent cytokine/chemokine activity.

##### KLF and NF-κB

Kruppel-like factor 4 (KLF4) is a zinc-finger transcription factor that plays a key role in cellular proliferation, differentiation, apoptosis and the generation of induced pluripotent stem (iPS) cells (McConnell and Yang 2010). Platelet-derived growth factor-BB (PDGF-BB) activates the deacetylation of KLF4 by promoting KLF4 dephosphorylation through the stimulation of the extracellular signal-regulated kinase (ERK) and PI3K/AKT pathways. Deacetylated KLF4 inhibits the differentiation makers of VSMCs through binding to G/C repressor element (Cherepanova et al. 2016; Salmon et al. 2012; Yu et al. 2011). On the contrary, Chen et al. find a novel protein, FAM172A, inhibits the transition of VSMCs from contractile to synthetic phenotype and increases atherosclerotic plaque stability by inhibiting KLF4 expression (Chen et al. 2021). Loss of KLF4 appears to promote the transition of VSMCs to an athero-protective phenotype with a > 60% decrease in the number of VSMC-derived macrophage-like and pro-inflammatory cells, but an increase in ACTA2 + VSMCs, which could increase the thickness of the fibrous cap. In addition, Ding et al. report that the upstream promoter of KLF4 contains a putative NF-κB binding site (Ding et al. 2016). NF-κB, a family of heterodimeric transcription factors, has long been considered a prototypical proinflammatory signaling pathway and modulates the expression of proinflammatory genes, including cytokines, chemokines and adhesion molecules (Lawrence 2009). Therefore, NF-κB selectively represses the potent VSMC coactivator MYOCD by cooperation with KLF4, eventually inhibiting the expression of VMSC differentiation genes (Liu et al. 2005; Yoshida et al. 2013). The abovementioned changes indicate that inhibition of KLF4 promotes plaque stabilization and alleviates the development of atherosclerotic lesions (Shankman et al. 2015). Interestingly, in contrast to KLF4, KLF15 has been proven to highly express in VSMCs under physiological conditions but decreases in atherosclerotic lesions (Lu et al. 2010). KLF15 could reduce the activity of NF-κB on inflammatory gene-associated promoters via direct repression of p300-dependent NF-κB acetylation (Lu et al. 2013). These findings have shown that the two KLF transcription factors have opposite effects on VSMC phenotypic switch.

##### OCT4

Octamer-binding transcription factor 4 (OCT4), the main stemness marker whose expression is associated with self-renewal and pluripotency maintenance of embryonic stem cells (Pesce and Scholer 2001), appears to promote an athero-protective phenotype. Loss of OCT4 results in phenotypic switch and subsequently weakens the plaque stability and accelerates the development of atherosclerotic lesions (Cherepanova et al. 2016). Although KLF4 and OCT4 play different roles in VSMC phenotypic transitions, Cherepanova et al. find that KLF4 appears to regulate OCT4 expression in VSMCs both in vitro and in vivo. Loss of KLF4 may lead to the downregulation of OCT4, which seems to be contrary to the anti-atherosclerosis effect of OCT4. However, activation of OCT4 in somatic cells appears to be more complex than simple KLF4 binding to the OCT4 promoter (Cherepanova et al. 2016). Other KLF family members, such as KLF2 or KLF5, may also activate OCT4 and subsequently regulate the early stages of VSMC phenotypic transitions. The interaction between transcription factors gives rise to the transcriptional regulatory network more complex.

##### NRF2, GAX, NFAT5 and PAX9

An essential modulator in protecting cells from oxidative stress, nuclear factor erythroid-2 related factor 2 (NRF2), is activated by choline, and then up-regulates the expression of its downstream proteins heme oxygenase-1 (HO-1) and NAD[P]H quinone oxidoreductase-1 (NQO-1), which could ameliorate the vascular remodeling via inhibiting the transformation of VSMCs to the synthetic phenotype (He et al. 2020). Besides, other transcription factors, such as growth arrest-specific homeobox (GAX) (Markmann et al. 2003) and Nuclear factor of activated T cells 5 (NFAT5) (Halterman et al. 2011), are also considered to alleviate the development of atherosclerotic lesions, loss of which is a major factor that contributes to atherosclerosis. On the contrary, paired box 9 (PAX9) has been known as promoting phenotypic switch and accelerating atherosclerotic lesions (Xu et al. 2020).

#### Pluripotency of phenotypic transformation

Recently, the emergence of lineage-tracing and scRNA-seq studies converts the well-established concept of the VSMC phenotypic switch and increases our ability to identify different phenotypes derived from VSMCs beyond staining for a few markers. During this process, VSMCs lose their surface markers and express the markers of VSMC-derived cells. More than 80% of phenotypically modulated VSMCs within plaque have previously not been detected and comprise 30% of the total cellular composition of the lesions. Both the function and number of VSMCs in plaque have been greatly underestimated (Alencar et al. 2020; Chen et al. 2020; Pan et al. 2020; Shankman et al. 2015).

##### KLF4

VSMCs are shown to the intrinsic plasticity in vivo and in vitro when exposed to free cholesterol. VSMCs could undergo phenotypic conversion, dedifferentiate and eventually express macrophage and fibroblast markers, which depends on the activation of KLF4 signaling via an unfolded protein response (UPR). Exposure to cholesterol activates all 3 pathways of the UPR, and the increase of KLF4 level has been known primarily via the Perk-eIF2α-Atf4 arm of the endoplasmic reticulum (ER) stress (Chattopadhyay et al. 2021; Shankman et al. 2015; Zhou et al. 2015). Although Li et al. report that activation of KLF4 contributes to macrophage-like VSMC phenotype modulation during atherogenesis (Li et al. 2021a), Alencar et al. suggest that instead of a terminal state of VSMCs switching to macrophages as originally postulated (Shankman et al. 2015), activation of Lgals3, typically considered as a marker for macrophages, appears to be an earlier transitional state during atherosclerosis. These so-called “pioneer cells” generate inflammatory, ECM and osteogenic cell states in atherosclerotic lesions (Alencar et al. 2020). Despite the presence of Lgals3, there are few derivatives of macrophage-like state in advanced atherosclerosis. Thus, under activation of UPR pathways and ER stress, contractile VSMCs dedifferentiate to a transitional state, just presence of Lgals3 as a marker of intermediate state but not macrophage state. In another study, with a significantly increased expression of KLF4 and its family members KLF2 and KLF5, contractile VSMCs are trans-differentiation into a mesenchymal stem cell (MSC)-like intermediate state that subsequently generates osteoblasts, chondrocytes, adipocytes and macrophages during the development of aortic aneurysms (Chen et al. 2020). Besides, Alencar et al. also find a significant reduction of Lgals3 VSMCs and high expression of VSMC contractile markers by targeting knockout KLF4, which promotes the stabilization of plaques. The role of transcription factor KLF4 in driving the phenotypic change of VSMCs has been found in other studies, Pan et al. demonstrate VSMC-derived “SEM” cells (stem cell, endothelial cell, monocyte), like the pioneer cells in the study of Alencar et al. (2020), Lgals3 + and multipotent, may transform into fibrochondrocyte-like, macrophage-like cells or return to the VSMC phenotype (Pan et al. 2020).

##### TCF21

TCF21, which has been reported associated with several cardiovascular diseases, regulates cell fate and differentiation specificity through an epithelial-mesenchymal transformation during cardiac development. In addition, it also plays a vital role in angiogenesis, endometriosis and autophagy (Ao et al. 2020). Wirka et al. observe that TCF21 is up-regulated during atherosclerotic lesions and promotes VSMCs to transform into a fibroblast-like phenotype, which is named as “fibromyocyte”. The loss of TCF21 inhibits the formation of protective fibrous caps by downregulation of fibroblast-like phenotype. Whether the derived cells of VSMCs eventually promote or inhibit atherosclerotic lesions needs to be further verified. Different from adopting multiple distinct cell phenotypes, Wirka et al. observe VSMC transition almost exclusively to fibromyocytes. The trajectories of gene expression move further away from a macrophage-like state and appear to exhibit a continuous trajectory from a contractile phenotype towards a fibroblast-like phenotype during disease’s progression (Wirka et al. 2019). Besides, integrative analyses suggest that the modulated VSMCs, “fibromyocytes”, may contain both the SEM cell and fibrochondrocyte clusters identified by Pan et al. (2020).

##### RUNX2, STAT and SLUG

Runt-related transcription factor 2 (RUNX2), the key osteogenic regulator for osteoblast differentiation and chondrocyte maturation, represses MYOCD-induced differentiation and promotes the osteogenic transformation of VSMCs via disrupting the formation of the MYOCD and SRF complex (Byon et al. 2008; Li et al. 2020b; Tanaka et al. 2008). Besides, Fractalkine (FKN), the only number of chemokine CX3G subgroup, binds to the membrane-specific receptor CX3CR1 on VSMCs in atherosclerotic lesions, subsequently activates the STAT3 signaling pathway to upregulation of RUNX2, eventually promotes RANK ligand and other osteogenic marker protein expression (Kakutani et al. 2015; Yang et al. 2020). Other transcription factors of the STAT family, such as STAT1, also up-regulate RUNX2 expression and exacerbate osteogenic differentiation in VSMCs (Li et al. 2020b). Cholesterol loading of VSMCs up-regulates the transcription factor SLUG expression. SLUG, which has been known to increase migratory properties, invasiveness and apoptotic resistance of epithelial cells during embryogenesis and cancer metastasis, is involved in VSMC displaying a macrophage-like phenotype in human carotid atherosclerotic lesions (Ledard et al. 2020). Cholesterol loading of VSMCs also inhibits MYOCD expression, which could convert VSMCs to a macrophage-like state by down-regulating the miR-143/145–MYOCD axis (Vengrenyuk et al. 2015).

##### NF-κB

In addition to transforming into the above phenotypes, VSMCs could differentiate into a phenotype that resembles lymphoid tissue organizers (LTO) by crosstalk of the classical RelA and alternative RelB NF-κB signaling pathways (Katakai et al. 2008; Luther et al. 2002; Mebius 2003). The interaction of classical and alternative NF-κB signaling pathways most likely forms RelA and RelB complex, which couples with target gene promoters to induce LTO phenotype (Lötzer et al. 2010).

#### VSMC-derived foam cells

Expect to known lipid-rich macrophages, multiple studies have also reported the presence of lipid-laden VSMCs. Wang et al. present striking evidence that 60–70% of total foam cells in atherosclerotic lesions derive from VSMCs, and not from macrophages (Wang et al. 2019b). This study also indicates that the expression of ATP-binding cassette transporter A1 (ABCA1), the rate-limiting exporter of cholesterol efflux, is lower in VSMC-derived foam cells compared with macrophage-derived foam cells. A decrease in cholesterol efflux leads to lipid retention and accelerates foam cell formation, which may explain why VSMCs become the main lineage of foam cells compared with macrophages. These foam cells have been detected mainly in advanced atherosclerotic plaque, as well as in early lesions. In addition, although mouse VSMCs express macrophage markers after lipid loading in vitro, their transcriptome remains closer to the VSMC state. Therefore, their ability of phagocytosis and endocytosis is weaker than macrophages (Vengrenyuk et al. 2015).

##### MYOCD and NFAT5

Cholesterol uptake and efflux are two fundamental processes of foam cell formation. MYOCD and SRF complex facilitates the binding of SRF onto the ABCA1 promoter and significantly increases its gene expression (Xia et al. 2021). Besides, MOYCD expression in VSMCs suppresses oxidized lipid uptake via downregulation of the scavenger receptor OLR-1, which mediates oxLDL uptake in VSMCs (Ackers-Johnson et al. 2015). In atherosclerosis, downregulation of MYOCD inhibits cholesterol efflux through down-regulating ABCA1 expression and promotes cholesterol uptake via upregulation of the scavenger receptor, eventually contributing to VSMC foam cell formation. Exposure to cholesterol also stimulates the nuclear translocation of NFAT5, a cholesterol-sensitive transcription factor in stabilizing responses of cells to hypertonic and biomechanical stress, which increases the expression of the ABCA1 and up-regulates cholesterol efflux from VSMCs. SMC-specific genetic ablation of NFAT5 promotes lipid accumulation at arteriosclerosis-prone sites (Kappert et al. 2021).

##### NF-κB, KLF4 and SLUG

Overexpression of cyclin G2 up-regulates the expression of nuclear NF-κB and promotes uptake and accumulation of lipids via inhibiting the enzymatic activity of PP2A, which plays an important role in the regulating NF-κB signaling pathway. Activation of PP2A reverses the effects of cyclin G2 on foam cell formation by suppressing the expression of NF-κB (Zhang et al. 2021). An increasing cholesterol loading induces upregulation of KLF4 expression. KLF4, a known repressor of MYOCD expression, could transform VSMCs into the foam cell phenotype by down-regulating the miR-143/145-MYOCD axis (Castiglioni et al. 2017). Other transcription factor, such as SLUG, also down-regulates genes related to cholesterol efflux and promotes a foam phenotype (Ledard et al. 2020).

### Proliferation and migration

Abnormal proliferation and migration of VSMCs are closely associated with the development of atherosclerotic lesions and play a critical role in restenosis after angioplasty of human coronary arteries (Charo and Taubman 2004). Compared with a very low proliferation rate in the healthy arterial wall, VSMC proliferation is regulated by several environmental factors during the progress of atherosclerotic lesions including growth factors and their receptors, vasoactive substances, inflammatory mediators, matrix component and cell–cell interactions (Owens et al. 2004). These factors stimulate VSMC proliferation partly by modulating downstream transcription factor-related signaling pathways. The interactions of these factors and signaling pathways are complex, and eventually regulate the cell cycle. The proliferation of VSMCs produces excessive fibrous tissue, forming the fibrous cap of the plaque and leading to intimal thickening and restenosis. Besides, VSMC migration also plays a crucial role in the pathological remodeling of the vascular walls during atherosclerosis. Vascular wall remodeling is characterized by an imbalance between ECM and MMPs. During the development of atherosclerotic lesions, activation of MMPs but not alteration of tissue inhibitors of matrix metalloproteinases (TIMPs) expression could cause the degradation of the ECM and promote VSMC migration, eventually leading to pathological remodeling and vascular restenosis (Cho and Reidy 2002; Ikeda and Shimada 2003; Newby and Zaltsman 2000).

#### KLF4, CHOP and KLF5

Induction of KLF4, a key suppressor of VSMC differentiation markers, also reduces the proliferation rate of VSMCs by regulating p21WAF1/CIP1 expression in concert with p53 without affecting apoptosis, and this process leads to the cell cycle stagnation in the G1/S and G2/M phases of post-mitosis (Godmann et al. 2009). Conditional KLF4 mutant mice exhibits enhanced proliferation following vascular injury (Yoshida et al. 2008). C/EBP homologous protein (CHOP), which has long been accepted as a vital trigger for ER stress-related apoptosis (Li et al. 2014), promotes VSMC proliferation by down-regulating KLF4, deletion of CHOP in atherosclerotic mice increases the KLF4 expression, and silencing KLF4 in CHOP‐deficient VSMCs in return restores proliferation (Zhou et al. 2015). Besides, regulating miR-92a expression by activating upstream regulators of both ROCK/STAT3 and MLCK/STAT3 signaling pathways could inhibit KLF4 expression and promote PDGF‐BB‐mediated proliferation and migration of VSMCs (Wang et al. 2019a). Different from inhibiting proliferation, KLF4 expression could promote the migration of VSMCs. Oxidized phospholipids activate the coordinate expression of a variety of ECM genes, including type VIII collagen in a KLF4 and specificity protein 1 (SP1)-dependent manner. Activation of type VIII collagen expression has been known for promoting the migration of VSMCs, indicating that KLF4 promotes VSMC migration (Cherepanova et al. 2009). Another KLF family number transcription factor KLF5, up-regulated in activated VSMCs and myofibroblasts in response to vascular injury (Nagai et al. 2005), also plays an important role in regulation of VSMC proliferation, migration and inflammation (Zhang et al. 2015; Zheng et al. 2011). oxLDL induces the expression of KLF5, which in turn increases miR-29a expression. The increased miR-29a inhibits FBW7/CDC4 expression by targeting its 3’UTR, subsequently increases KLF5 stability by reducing KLF5 ubiquitination and thereby forms a positive oxLDL-KLF5-miR-29a-FBW7/CDC4-KLF5 feedback loop to enhance VSMC proliferation and promote atherogenesis (Zheng et al. 2018). Besides, KLF5 is required for PDGF-induced cyclin D1 expression, which could accelerate the cell cycle to enhance proliferation (Martin-Garrido et al. 2013). Weng et al. report that VSMC proliferation and migration could be up-regulated by lncRNA LINC01123, which down-regulates miR-1277-5p expression, a negative regulator of KLF5 expression (Weng et al. 2021). Li et al. also find that VSMCs enrich lncRNA SMILR, which contributes to the progression of VSMC proliferation by sponging miR-10b-3p to regulate the expression of KLF5 (Li et al. 2020a).

#### STAT

Activated STAT, a downstream target of janus kinase (JAK), could transport into the nucleus to combine with specific regulatory sequences to activate or suppress transcription of target genes, which are related to diverse cardiac pathophysiologic progress, including apoptosis, proliferation, immune regulation and inflammation (Bolli et al. 2003). Transcription factor STAT3 becomes transcriptionally activated by tyrosine phosphorylation and then translocates into the nucleus (Akira et al. 1994). In addition to inhibiting KLF4 expression and promoting VSMC proliferation and migration, STAT3 activates LOXL1-AS1 transcription in VSMCs, which could, in turn, elevate STAT3 expression by sponging miR-515-5p. LOXL1-AS1/miR-515-5p/STAT3 positive feedback loop facilitates VSMC proliferation and migration (Xie et al. 2020). Nicotine stimulates the nuclear translocation of STAT3 and increases its binding to the AKT promoter region. STAT3 inhibition attenuates nicotine-induced VSMC migration and proliferation via AKT/mTOR/MMP2 signaling pathway (Xu et al. 2019). Besides, STAT1 provides a platform for cross-talk between interferon γ (IFNγ) and lipopolysaccharides (LPS), this cross-talk results in STAT1-dependent VSMC activation, increases expression of ICAM-1 and IP-10 (Sikorski et al. 2011a), and eventually promotes VSMC proliferation and migration (Sikorski et al. 2011b). In mice, total knockdown STAT1 and STAT3 genes prevent atherosclerotic lesions (Agrawal et al. 2007; Gharavi et al. 2007; Lim et al. 2008) by inhibiting PDGF-BB-induced VSMC proliferation and migration (Albasanz-Puig et al. 2011; Shigetoshi et al. 1999; Torella et al. 2007). Pharmacological inhibition of STAT1 and STAT3 also reduces lesion size and neointimal hyperplasia (Daniel et al. 2012; Torella et al. 2007).

#### NF-κB, NFAT5 and PTEN

RelA, also known as p65, is the most common member of NF-κB family and is activated by NF-κB canonical pathway (Fiordelisi et al. 2019). Treated with miR-520c-3p mimics attenuates VSMC proliferation and migration via suppression of RelA. Overexpression of RelA, in turn, partially alleviates the suppression effects of miR-520c-3p (Wang et al. 2021). NFAT5, which DNA-binding domain is highly conserved with the Rel family protein NF-κB (Aramburu and Lopez-Rodriguez 2019), positively regulates PDGF-BB and serum-induced VSMC migration but not proliferation (Halterman et al. 2011). Yeh et al. find Vanadium derivatives, VOSO4 and NaVO3, selectively activate the ROS-triggering p38 signaling to induce NF-κB-mediated IL-6 production, which eventually leads to VSMC synthetic differentiation, proliferation and migration (Yeh et al. 2019). Li et al. report that miR‐141‐5p inhibits the abnormal proliferation and migration during the pathogenesis of atherosclerosis by repressing the HMGB1/NF‐κB pathway (Li et al. 2021b). These studies demonstrate that NF‐κB plays a significant role in regulating VSMC proliferation and migration. Overexpression of PTEN directly inhibits angiotensin II (ANG II)-induced monocyte chemoattractant protein-1 (MCP-1) and NF-κB expression in cultured VSMCs (Koide et al. 2007). MCP-1, a potent chemoattractant for VSMCs, is mediated by ERK and NF-κB (Chen et al. 1998; Hernandez-Presa et al. 1997). Either stimulation of ANG II (Valente et al. 2012) or genetic reduction of PTEN could specifically activate NF-κB (Lamers et al. 2012), and subsequently lead to neointima formation and atherosclerotic progression (Furgeson et al. 2010). Besides, PTEN exerts inhibition of VSMC proliferation and migration through down-regulating growth factor-induced activation of both AKT and P70S6K (Huang and Kontos 2002; Zhang et al. 2019). Whereas, vascular injury results in PTEN inactivation and constitutive expression of MMP-9 through a PI3K/AKT dependent NF-κB signaling, which stimulates the migration of VSMCs (Chandrasekar et al. 2006). Loss of nuclear PTEN also results in a reduction of SRF to combine the differentiated phenotype gene promoters but enhances SRF activity on target gene promoters involved in VSMC proliferation (Horita et al. 2016).

#### FOXO1/3, PAX9 and OCT4

Activation of the serine/threonine kinase AKT could phosphorylate numerous downstream target proteins, such as transcription factor forkhead box O3 (FOXO3). Phosphorylated FOXO3, the main forkhead transcription factor related to the regulation of the cell cycle, DNA repair, defense against oxidative stress and longevity, is rendered inactive by exclusion from the nucleus into the cytoplasm and rapid degradation to regulate VSMC proliferation and migration (Fasano et al. 2019). Angiogenic immediate early gene CYR61, a target gene of FOXO3, is highly expressed in human atherosclerotic plaque, and FOXO3 is a negative transcriptional regulator of CYR61. Phosphorylated FoxO3 alleviates the inhibition of CYR61 and promotes CYR61-dependent proliferation and migration (Lee et al. 2007). Besides, phosphorylation of FOXO3 also plays a central role in mediating VSMC migration by up-regulating MMP-2 and inhibiting TIMP-2 (Wang et al. 2015). Interestingly, FOXO3 activation markedly induces MMP-13 expression and secretion from VSMCs, and MMP-13 is a direct FOXO3 transcriptional target in VSMCs. FOXO3 reduces TIMP-1 to 3 expression at both RNA and protein levels, which could further increase MMP-13 activity and promote VSMC migration (Yu et al. 2018). Ma et al. confirm that aberrant expression of circRNA hsa_circ_0030042 induces FOXO1 expression and then promotes VSMC proliferation and migration. Silencing of FOXO1 eventually inhibits VSMC proliferation and migration (Ma et al. 2022). Besides, overexpression of transcription factor PAX9, a well-known transcription factor involved in the human dentitional development and embryonic development of various tissues and organs, increases the levels of MMP-2 and MMP-9 protein expression (Xu et al. 2020), OCT4 could promote expression of MMP-3 and MMP-13 (Yang et al. 2015), all these matrix metalloproteinases have been shown to promote the migration of VSMCs.

#### MYOCD, NRF2, MEF2C and TR3

Inhibition of VSMC proliferation and migration by transcription factors alleviates atherogenesis. Unfortunately, the expression of these transcription factors is mostly down-regulated during lesion deterioration. MYOCD, an anti-atherosclerotic factor, inhibits VSMC proliferation via two pathways, one is to decrease p65-mediated expression of target genes by directly binding to the p65 subunit (Tang et al. 2008), the other is to induce expression of miR-1, which is reversed by miR-1 inhibitors (Chen et al. 2011). MYOCD also inhibits VSMC migration. There are no intimal VSMCs in healthy mice, however, the presence of intimal VSMCs, accompanied by a decrease in MYOCD expression in response to atherosclerotic plaques, has been considered evidence that MYOCD regulates VSMC migration from the arterial media (Talasila et al. 2013). Besides, inhibited expression of MYOCD by upregulation of IQ-domain GTPase-activating protein 1 (IQGAP1) in human VSMCs dramatically enhances VSMC migration and rearrangement (Huang et al. 2014). NRF2, a transcription factor that regulates the glutathione and thioredoxin antioxidant systems and detoxification of exogenous and endogenous products, represents a crucial regulator of the oxidative stress and anti-inflammatory process (Ahmed et al. 2017). Ko et al. find that NRF2, which could be activated by p38 mitogen-activated protein kinase, induces the activation of the anti-inflammatory signaling pathway NRF2/HO-1, and then inhibits the proliferation and migration of VSMCs in mouse aorta (Ko et al. 2020). Jiang et al. demonstrate that Sulfiredoxin1, an endogenous antioxidant protein, inhibits PDGF-BB-induced VSMC proliferation and migration via enhancing the activation of NRF2/ARE signaling (Jiang et al. 2022a). In addition to the NRF2 signaling pathway, the expression of myocyte enhancer factor 2C (MEF2C) inhibits VSMC proliferation and migration. Overexpression of miR-448, miR-135b-5p and miR-499a-3p could down-regulate the MEF2C mRNA and protein expression in VSMCs and ultimately stimulate VSMC proliferation and migration isolated from coronary atherosclerotic plaque compared with normal coronary artery tissue (Xu et al. 2015; Zhang et al. 2017). Besides, transcription factor TR3, which has been known to control of biological activities of survival and death of cancer cells and tumors, is expressed in atherosclerotic lesions to protect the vessel wall from excessive VSMC proliferation. Lesions in TR3 over-expressing transgenic mice were fivefold smaller than in isogenic wild-type mice (Arkenbout et al. 2002).

#### HIF-1, SP1, TCF21 and AP1

In addition to the above-mentioned, other transcription factors also up-regulate and promote VSMC proliferation and migration in atherosclerotic lesions. Overexpression of hypoxia-inducible transcription factor-1 (HIF-1), a ubiquitous transcription factor that regulates cellular oxygenation and metabolic adaptation to hypoxic states, up-regulates macrophage migration inhibitory factor (MIF) expression in VSMCs. The hypoxia-induced MIF expression plays a vital role in increased VSMC proliferation and migration in an autocrine and paracrine manner. Knockdown of HIF-1 prevents any hypoxia-induced migration (Fu et al. 2010). Besides, VSMC proliferation and migration may also be induced by transcription factor SP1. SP1, regulation of various cellular processes through regulating cell cycle signaling pathway genes, tissue-specific genes and housekeeping genes (Jiang et al. 2022b), is required for PDGF-BB-inducible TNF-related apoptosis-inducing ligand (TRAIL) transcription, which might promote VSMC proliferation and migration (Azahri et al. 2012; Zhang 2020). There is also an SP1-binding site on the promoter of MMP-9, the binding activity of SP1 to the MMP-9 promoter region also increases the PDGF-induced VSMC proliferation and migration (Shin et al. 2018). Besides, the expression of transcription factor TCF21 shows a time-dependent increase in proliferation, migration and fibrous cap formation (Nurnberg et al. 2015). The VSMC proliferation could also be activated via the action of activator protein 1 (AP1), which has been implicated in the regulation of cellular proliferation in vitro and after arterial injury in vivo (Rivard et al. 2000).

### Apoptosis

The existence of VSMCs plays an indispensable role in maintaining the stability of fibrous cap, which primarily consists of VSMCs and a comparatively dense ECM made of elastin, proteoglycans and collagen. The apoptosis of VSMCs may aggravate the atherosclerotic plaque via decreasing collagen production, consequently thinning the protective fibrous cap and leading to the accumulation of cell debris (Clarke et al. 2006). Moreover, the apoptosis of VSMC itself has also been known as a factor to induce plaque vulnerability (Clarke and Bennett 2006; Clarke et al. 2006). Suppression of the endogenous VSMC apoptosis in atherogenesis promotes a relatively thicker fibrous cap and smaller atherosclerotic plaque volume, demonstrating the beneficial effect of VSMC survival during atherogenesis.

#### CHOP

Various inducers have been identified to increase CHOP levels. Despite the beneficial effect of a transient UPR, prolonged ER stress-induced CHOP expression plays a critical role in VSMC apoptosis both in vitro and vivo (Li et al. 2014; Tabas and Ron 2011). ER stress occurs in atherosclerotic plaque, particularly in the advanced atherosclerotic stage (Myoishi et al. 2007). However, the specific mechanism of ER stress-induced CHOP expression in VSMCs needs further exploration. Under an atherosclerotic situation, one mechanism of CHOP-induced apoptosis involves transcriptional downregulation of the BCL-2 protein family, therefore accelerating apoptosis of VSMCs (McCullough et al. 2001). CHOP could also induce the transcription of BIM, which plays a crucial role in mitochondrial-mediated apoptosis (Puthalakath et al. 2007). Besides, CHOP-induced apoptosis involves a calcium signaling pathway. CHOP-dependent activation of the ER oxidase 1 alpha triggers calcium release by activating the ER calcium channel (Li et al. 2009), which triggers several downstream calcium-dependent apoptosis pathways.

#### FOXO3

FOXO3 activation leads to VSMC apoptosis and increases necrotic core area and unstable fibrous cap compared with the non-atherosclerotic vessel (Allard et al. 2008). The expression of AKT1 inhibits VSMC apoptosis via identifying the transcriptional targets of FOXO3 and making FOXO3 phosphorylation (Tucka et al. 2014). During atherosclerotic progression, phosphorylated FOXO3 expression is reduced in intimal VSMCs of human plaque (Allard et al. 2008), implying that dephosphorylated FOXO3 promotes VSMC apoptosis in atherosclerotic plaque. Apoptotic protease activating factor-1 (APAF1), a FOXO3 target gene, regulates FOXO3-mediated apoptosis by binding FOXO3 to its promoter. Inhibition of FOXO3 dependent APAF1 expression could delay VSMC apoptosis in vitro. Besides, FOXO3-induced apoptosis is mediated, in part, by MMP-13-induced ECM degradation (Yu et al. 2018). The ECM proteins, including fibronectin and N-cadherin, protect VSMCs against apoptosis, both of which may be targets of FOXO3 and MMPs (Lee et al. 2008; Williams et al. 2010). Moreover, MMP-13 is a direct FOXO3 transcriptional target in VSMCs and MMP-13-specific inhibitors reduce FOXO3-mediated apoptosis (Yu et al. 2018).

#### PTEN, KLF5 and SP1

PTEN induces VSMC apoptosis by inhibiting both basal and growth factor-induced cell survival pathways, and overexpression of PTEN blocks PI3K/AKT signaling pathway and enhances caspase-3 cleavage in VSMCs, both of which eventually promote VSMC apoptosis (Huang and Kontos 2002). Whereas not all transcription factors promote VSMC apoptosis, the KLF5 overexpression could inhibit VSMC apoptosis by reversing the inhibition of BCL-2 protein expression in atherosclerotic lesions (Li et al. 2020a). Zhang also reports that the upregulation of circRNA circPTPRA in serum samples of atherosclerosis could inhibit VSMC apoptosis via up-regulating SP1 signaling axis (Zhang 2020).

### Calcification

A significant characteristic of advanced atherosclerosis is vascular calcification, the calcium deposition gradually reduces elasticity and compliance of the vascular wall (Trion and van der Laarse 2004). Calcification could begin at the initial stage, accelerate during the atherosclerotic development, and eventually promote plaque destabilization (Demer and Tintut 2011). During this process, the differentiated VSMCs undergo osteogenic transition, this transformation has been confirmed as the major cause of vascular calcification (Hayashi et al. 2006).

#### RUNX2 and FOXO1/3

Many transcription factors have been linked to an increased prevalence of vascular calcification. RUNX2 plays an essential role in regulating vascular calcification during atherosclerosis (Sun et al. 2012). One mechanism of RUNX2-induced calcification involves activation of AKT signaling under PTEN deficiency, which promotes RUNX2 upregulation and VSMC calcification (Byon et al. 2008). RUNX2 knockdown significantly inhibits VSMC calcification but doesn’t affect proliferation of the PTEN-deficient VSMCs, suggesting that activation of AKT promotes RUNX2-dependent VSMC calcification (Deng et al. 2015). Furthermore, this blockage has no effect on VSMC apoptosis (Byon et al. 2008). In another study, Lino et al. also find discoidin domain receptor 1 (DDR1) mediates vascular calcification in an animal model of diabetic atherosclerosis via activation of AKT signaling to induce RUNX2 activity (Lino et al. 2018). Besides, AKT activation promotes cytosolic translocation of FOXO1/FOXO3, which is also associated with upregulation of RUNX2. FOXO1/3 knockdown inhibits RUNX2 ubiquitination and promotes VSMC calcification (Deng et al. 2015). Activation of other transcription factors, such as STAT1 and KLF4, could directly bind to the RUNX2 promoter and up-regulate RUNX2 transcriptional activity, leading to aggravating calcification in VSMCs (Li et al. 2020b; Zhu et al. 2019).

#### NF-κB, CHOP and NRF2

Zhao et al. report that activation of NF-κB promotes inorganic phosphate-induced calcification and reduces pyrophosphate (an inhibitor of calcification) efflux to the ECM via down-regulating ankylosis protein homolog (ANKH), a transmembrane protein controlling pyrophosphate secretion (Zhao et al. 2012). In another study, upregulation of NF-κB-dependent MSX2 induces the expression of alkaline phosphatase (ALP), a key molecule in aggravating vascular calcification (Lee et al. 2010). Transcription factor CHOP has been known as a pro-apoptotic signaling molecule. Activation of the ER stress induces upregulation of the PERK-eIF2α-ATF4-CHOP axis in VSMCs. CHOP knockdown blocks CHOP protein level and results in a significant reduction of vascular calcification (Masuda et al. 2013). Hydrogen sulfide, known to reduce cytokine production and ameliorate oxidative stress, attenuates circulating calciprotein particles (CPP)-induced VSMC calcification model in vitro by increased expression of NQO1 via activation of NRF2. Silencing of NRF2 significantly decreases the expression of both the NRF2 and NQO1 gene and eventually aggravates the extent of calcification (Aghagolzadeh et al. 2017).

## Conclusion and perspective

Transcription factors play an important role in VSMC regulation during atherosclerotic lesions. While previous studies have proved that transcription factors and their target genes can shape complex transcriptional regulatory networks, our understanding of the complexity and vulnerability of these networks is still far from enough. Recently, the application of new biotechnologies provides us with new insights in identifying transcription factors. The links between transcription factors and VSMCs have established a new field for the diagnosis and therapy of atherosclerosis. A deeper understanding of transcriptional regulatory mechanisms will lead to the identification of novel therapeutic targets for atherosclerosis and the development of target-based new anti-atherosclerotic drugs. Besides, research on VSMCs should continue to be in-depth by combining with the regulation of transcription factors. A comprehensive understanding of VSMCs will alleviate the progression of atherosclerosis, which has been proved as a remarkably promising direction.

## Data Availability

Not applicable.

## Abbreviations

AS: Atherosclerosis
VSMCs: Vascular smooth muscle cells
LDL: Low-density lipoprotein
oxLDL: Oxidized low-density lipoprotein
ECM: Extracellular matrix
MMPs: Matrix metalloproteinases
MYOCD: Myocardin
SRF: Serum response factor
TCF21: Transcription factor 21
PTEN: Phosphatase and tensin homolog
PI3K: Phosphoinositide 3-kinase
AKT: Protein kinase B
mTOR: Mammalian target of rapamycin
NF-κB: Nuclear factor kappa-B
KLF: Kruppel-like factor
iPS: Induced pluripotent stem
PDGF-BB: Platelet-derived growth factor-BB
ERK: Extracellular signal-regulated kinase
OCT4: Octamer-binding transcription factor 4
NRF2: Nuclear factor erythroid-2 related factor 2
HO-1: Heme oxygenase-1
NQO-1: NAD[P]H quinone oxidoreductase-1
GAX: Growth arrest-specific homeobox
NFAT5: Nuclear factor of activated T cells 5
PAX9: Paired box 9
UPR: Unfolded protein response
ER: Endoplasmic reticulum
MSC: Mesenchymal stem cell
SEM: Stem cell, endothelial cell, monocyte
RUNX2: Runt-related transcription factor 2
FKN: Fractalkine
LTO: Lymphoid tissue organizers
ABCA1: ATP-binding cassette transporter A1
TIMPs: Tissue inhibitors of matrix metalloproteinases
CHOP: C/EBP homologous protein
SP1: Specificity protein 1
JAK: Janus kinase
IFNγ: Interferon γ
LPS: Lipopolysaccharides
ANG II: Angiotensin II
MCP-1: Monocyte chemoattractant protein-1
FOXO: Forkhead box O
IQGAP1: IQ-domain GTPase-activating protein 1
MEF2C: Myocyte enhancer factor 2C
HIF-1: Hypoxia-inducible transcription factor-1
MIF: Macrophage migration inhibitory factor
TRAIL: TNF-related apoptosis-inducing ligand
AP1: Activator protein 1
APAF1: Apoptotic protease activating factor 1
DDR1: Discoidin domain receptor 1
ANKH: Ankylosis protein homolog
ALP: Alkaline phosphatase
CPP: Calciprotein particles
BCL-2: B-cell lymphoma-2
Col 8A1: Collagen type VIII alpha 1
VCAM1: Vascular cell adhesion molecule 1
ICAM1: Intercellular cell adhesion molecule 1
LOX-1: Lectin-like oxidized low-density-lipoprotein receptor-1
CDK1: Cyclin-dependent kinases 1
IL-6: Interleukin-6
PLIN2: Perilipin 2
CXCL: CXC motif ligand
SMMLCK: Smooth muscle myosin light chain kinase
ERO1-α: ER oxidoreductase 1-alpha
SOD1: Superoxide dismutase 1
FABP3: Fatty acid binding protein 3
PPARD: Peroxisome proliferator activated receptor delta
LOXL1: Lysyl oxidase like protein 1
IP-10: Interferon γ inducible protein-10
PCNA: Proliferating cell nuclear antigen
RANKL: Receptor or activator of NF-κB ligand
Col IA1: Collagen type I alpha 1
COX-2: Cyclooxygenase-2
ABCG1: ATP-binding cassette transporter G1
TFs: Transcription factors

## References

[CR1] Ackers-Johnson M (2015). Myocardin regulates vascular smooth muscle cell inflammatory activation and disease. Arterioscler Thromb Vasc Biol.

[CR2] Aghagolzadeh P (2017). Hydrogen sulfide attenuates calcification of vascular smooth muscle cells via KEAP1/NRF2/NQO1 activation. Atherosclerosis.

[CR3] Agrawal S (2007). Signal transducer and activator of transcription 1 is required for optimal foam cell formation and atherosclerotic lesion development. Circulation.

[CR4] Ahmed SM (2017). Nrf2 signaling pathway: pivotal roles in inflammation. Biochim Biophys Acta Mol Basis Dis.

[CR5] Akira S (1994). Molecular cloning of APRF, a novel IFN-stimulated gene factor 3 p91-related transcription factor involved in the gp130-mediated signaling pathway. Cell.

[CR6] Albasanz-Puig A (2011). Oncostatin M is expressed in atherosclerotic lesions: a role for Oncostatin M in the pathogenesis of atherosclerosis. Atherosclerosis.

[CR7] Alencar GF (2020). Stem cell pluripotency genes Klf4 and Oct4 regulate complex SMC phenotypic changes critical in late-stage atherosclerotic lesion pathogenesis. Circulation.

[CR8] Alexander MR, Owens GK (2012). Epigenetic control of smooth muscle cell differentiation and phenotypic switching in vascular development and disease. Annu Rev Physiol.

[CR9] Allahverdian S (2018). Smooth muscle cell fate and plasticity in atherosclerosis. Cardiovasc Res.

[CR10] Allard D, Figg N, Bennett MR, Littlewood TD (2008). Akt regulates the survival of vascular smooth muscle cells via inhibition of FoxO3a and GSK3. J Biol Chem.

[CR11] Ao X (2020). TCF21: a critical transcription factor in health and cancer. J Mol Med (berl).

[CR12] Aramburu J, Lopez-Rodriguez C (2019). Regulation of inflammatory functions of macrophages and T lymphocytes by NFAT5. Front Immunol.

[CR13] Arkenbout EK (2002). Protective function of transcription factor TR3 orphan receptor in atherogenesis: decreased lesion formation in carotid artery ligation model in TR3 transgenic mice. Circulation.

[CR14] Azahri NS, Di Bartolo BA, Khachigian LM, Kavurma MM (2012). Sp1, acetylated histone-3 and p300 regulate TRAIL transcription: mechanisms of PDGF-BB-mediated VSMC proliferation and migration. J Cell Biochem.

[CR15] Basatemur GL (2019). Vascular smooth muscle cells in atherosclerosis. Nat Rev Cardiol.

[CR16] Bennett MR, Sinha S, Owens GK (2016). Vascular smooth muscle cells in atherosclerosis. Circ Res.

[CR17] Bentzon JF, Otsuka F, Virmani R, Falk E (2014). Mechanisms of plaque formation and rupture. Circ Res.

[CR18] Bolli R, Dawn B, Xuan YT (2003). Role of the JAK-STAT pathway in protection against myocardial ischemia/reperfusion injury. Trends Cardiovasc Med.

[CR19] Byon CH (2008). Oxidative stress induces vascular calcification through modulation of the osteogenic transcription factor Runx2 by AKT signaling. J Biol Chem.

[CR20] Castiglioni S (2017). ABCA1 and HDL3 are required to modulate smooth muscle cells phenotypic switch after cholesterol loading. Atherosclerosis.

[CR21] Chandrasekar B (2006). Interleukin-18-induced human coronary artery smooth muscle cell migration is dependent on NF-kappaB- and AP-1-mediated matrix metalloproteinase-9 expression and is inhibited by atorvastatin. J Biol Chem.

[CR22] Charo IF, Taubman MB (2004). Chemokines in the pathogenesis of vascular disease. Circ Res.

[CR23] Chattopadhyay A (2021). Cholesterol-induced phenotypic modulation of smooth muscle cells to macrophage/fibroblast-like cells is driven by an unfolded protein response. Arterioscler Thromb Vasc Biol.

[CR24] Chen XL (1998). Angiotensin II induces monocyte chemoattractant protein-1 gene expression in rat vascular smooth muscle cells. Circ Res.

[CR25] Chen J, Kitchen CM, Streb JW, Miano JM (2002). Myocardin: a component of a molecular switch for smooth muscle differentiation. J Mol Cell Cardiol.

[CR26] Chen J (2011). Induction of microRNA-1 by myocardin in smooth muscle cells inhibits cell proliferation. Arterioscler Thromb Vasc Biol.

[CR27] Chen PY (2020). (msc) smooth muscle cell reprogramming in aortic aneurysms. Cell Stem Cell.

[CR28] Chen MY (2021). Deletion of Fam172a accelerates advanced atherosclerosis and induces plaque instability. Atherosclerosis.

[CR29] Cherepanova OA (2009). Oxidized phospholipids induce type VIII collagen expression and vascular smooth muscle cell migration. Circ Res.

[CR30] Cherepanova OA (2016). Activation of the pluripotency factor OCT4 in smooth muscle cells is atheroprotective. Nat Med.

[CR31] Cho A, Reidy MA (2002). Matrix metalloproteinase-9 is necessary for the regulation of smooth muscle cell replication and migration after arterial injury. Circ Res.

[CR32] Clarke M, Bennett M (2006). The emerging role of vascular smooth muscle cell apoptosis in atherosclerosis and plaque stability. Am J Nephrol.

[CR33] Clarke MC (2006). Apoptosis of vascular smooth muscle cells induces features of plaque vulnerability in atherosclerosis. Nat Med.

[CR34] Clarke MC (2008). Chronic apoptosis of vascular smooth muscle cells accelerates atherosclerosis and promotes calcification and medial degeneration. Circ Res.

[CR35] Daniel JM (2012). Inhibition of STAT3 signaling prevents vascular smooth muscle cell proliferation and neointima formation. Basic Res Cardiol.

[CR36] Demer L, Tintut Y (2011). The roles of lipid oxidation products and receptor activator of nuclear factor-kappaB signaling in atherosclerotic calcification. Circ Res.

[CR37] Deng L (2015). Inhibition of FOXO1/3 promotes vascular calcification. Arterioscler Thromb Vasc Biol.

[CR38] Ding Y (2016). AMP-activated protein kinase alpha 2 deletion induces VSMC phenotypic switching and reduces features of atherosclerotic plaque stability. Circ Res.

[CR39] Fasano C (2019). FOXO3a from the nucleus to the mitochondria: a round trip in cellular stress response. Cells.

[CR40] Fiordelisi A (2019). NFkappaB is a key player in the crosstalk between inflammation and cardiovascular diseases. Int J Mol Sci.

[CR41] Fu H (2010). Hypoxia stimulates the expression of macrophage migration inhibitory factor in human vascular smooth muscle cells via HIF-1alpha dependent pathway. BMC Cell Biol.

[CR42] Furgeson SB (2010). Inactivation of the tumour suppressor, PTEN, in smooth muscle promotes a pro-inflammatory phenotype and enhances neointima formation. Cardiovasc Res.

[CR43] Gharavi NM (2007). Role of the Jak/STAT pathway in the regulation of interleukin-8 transcription by oxidized phospholipids in vitro and in atherosclerosis in vivo. J Biol Chem.

[CR44] Godmann M (2009). Kruppel-like factor 4, a “pluripotency transcription factor” highly expressed in male postmeiotic germ cells, is dispensable for spermatogenesis in the mouse. Mech Dev.

[CR45] Goikuria H, Vandenbroeck K, Alloza I (2018). Inflammation in human carotid atheroma plaques. Cytokine Growth Factor Rev.

[CR46] Halterman JA (2011). Nuclear factor of activated T cells 5 regulates vascular smooth muscle cell phenotypic modulation. Arterioscler Thromb Vasc Biol.

[CR47] Hayashi K, Nakamura S, Nishida W, Sobue K (2006). Bone morphogenetic protein-induced MSX1 and MSX2 inhibit myocardin-dependent smooth muscle gene transcription. Mol Cell Biol.

[CR48] He X (2020). Activation of M3AChR (Type 3 Muscarinic Acetylcholine Receptor) and Nrf2 (Nuclear Factor Erythroid 2-Related Factor 2) signaling by choline alleviates vascular smooth muscle cell phenotypic switching and vascular remodeling. Arterioscler Thromb Vasc Biol.

[CR49] Hernandez-Presa M (1997). Angiotensin-converting enzyme inhibition prevents arterial nuclear factor-kappa B activation, monocyte chemoattractant protein-1 expression, and macrophage infiltration in a rabbit model of early accelerated atherosclerosis. Circulation.

[CR50] Horita H (2016). Nuclear PTEN functions as an essential regulator of SRF-dependent transcription to control smooth muscle differentiation. Nat Commun.

[CR51] Huang J, Kontos CD (2002). Inhibition of vascular smooth muscle cell proliferation, migration, and survival by the tumor suppressor protein PTEN. Arterioscler Thromb Vasc Biol.

[CR52] Huang X (2014). IQGAP1 promotes the phenotypic switch of vascular smooth muscle by myocardin pathway: a potential target for varicose vein. Int J Clin Exp Pathol.

[CR53] Huang J (2015). Myocardin is required for maintenance of vascular and visceral smooth muscle homeostasis during postnatal development. Proc Natl Acad Sci U S A.

[CR54] Huff MW, Pickering JG (2015). Can a vascular smooth muscle-derived foam-cell really change its spots?. Arterioscler Thromb Vasc Biol.

[CR55] Ikeda U, Shimada K (2003). Matrix metalloproteinases and coronary artery diseases. Clin Cardiol.

[CR56] Jiang H, Zhao Y, Feng P, Liu Y (2022). Sulfiredoxin-1 inhibits PDGF-BB-induced vascular smooth muscle cell proliferation and migration by enhancing the activation of Nrf2/ARE signaling. Int Heart J.

[CR57] Jiang JF (2022). Role of Sp1 in atherosclerosis. Mol Biol Rep.

[CR58] Kakutani Y (2015). Oncostatin M promotes osteoblastic differentiation of human vascular smooth muscle cells through JAK3-STAT3 pathway. J Cell Biochem.

[CR59] Kappert L (2021). Loss of Nfat5 promotes lipid accumulation in vascular smooth muscle cells. Faseb j.

[CR60] Katakai T (2008). Organizer-like reticular stromal cell layer common to adult secondary lymphoid organs. J Immunol.

[CR61] Ko WC, Shieh JM, Wu WB (2020). P38 MAPK and Nrf2 activation mediated naked gold nanoparticle induced heme oxygenase-1 expression in rat aortic vascular smooth muscle cells. Arch Med Res.

[CR62] Koide S (2007). PTEN reduces cuff-induced neointima formation and proinflammatory cytokines. Am J Physiol Heart Circ Physiol.

[CR63] Lamers D (2012). Differential impact of oleate, palmitate, and adipokines on expression of NF-kappaB target genes in human vascular smooth muscle cells. Mol Cell Endocrinol.

[CR64] Lawrence T (2009). The nuclear factor NF-kappaB pathway in inflammation. Cold Spring Harb Perspect Biol.

[CR65] Ledard N (2020). Slug, a cancer-related transcription factor, is involved in vascular smooth muscle cell transdifferentiation induced by platelet-derived growth factor-BB during atherosclerosis. J Am Heart Assoc.

[CR66] Lee SH, Hungerford JE, Little CD, Iruela-Arispe ML (1997). Proliferation and differentiation of smooth muscle cell precursors occurs simultaneously during the development of the vessel wall. Dev Dyn.

[CR67] Lee HY (2007). Forkhead transcription factor FOXO3a is a negative regulator of angiogenic immediate early gene CYR61, leading to inhibition of vascular smooth muscle cell proliferation and neointimal hyperplasia. Circ Res.

[CR68] Lee HY (2008). Forkhead factor, FOXO3a, induces apoptosis of endothelial cells through activation of matrix metalloproteinases. Arterioscler Thromb Vasc Biol.

[CR69] Lee HL, Woo KM, Ryoo HM, Baek JH (2010). Tumor necrosis factor-alpha increases alkaline phosphatase expression in vascular smooth muscle cells via MSX2 induction. Biochem Biophys Res Commun.

[CR70] Li G (2009). Role of ERO1-alpha-mediated stimulation of inositol 1,4,5-triphosphate receptor activity in endoplasmic reticulum stress-induced apoptosis. J Cell Biol.

[CR71] Li Y (2014). New insights into the roles of CHOP-induced apoptosis in ER stress. Acta Biochim Biophys Sin (shanghai).

[CR72] Li H (2020). SMILR aggravates the progression of atherosclerosis by sponging miR-10b-3p to regulate KLF5 expression. Inflammation.

[CR73] Li P (2020). Loss of PARP-1 attenuates diabetic arteriosclerotic calcification via Stat1/Runx2 axis. Cell Death Dis.

[CR74] Li Y (2021). RNA Splicing of the Abi1 Gene by MBNL1 contributes to macrophage-like phenotype modulation of vascular smooth muscle cell during atherogenesis. Cell Prolif.

[CR75] Li Y (2021). miR-141-5p suppresses vascular smooth muscle cell inflammation, proliferation, and migration via inhibiting the HMGB1/NF-κB pathway. J Biochem Mol Toxicol.

[CR76] Libby P, Ridker PM, Hansson GK (2011). Progress and challenges in translating the biology of atherosclerosis. Nature.

[CR77] Lim WS (2008). Signal transducer and activator of transcription-1 is critical for apoptosis in macrophages subjected to endoplasmic reticulum stress in vitro and in advanced atherosclerotic lesions in vivo. Circulation.

[CR78] Lino M (2018). Diabetic vascular calcification mediated by the collagen receptor discoidin domain receptor 1 via the phosphoinositide 3-kinase/Akt/Runt-related transcription factor 2 signaling axis. Arterioscler Thromb Vasc Biol.

[CR79] Liu Y (2005). Kruppel-like factor 4 abrogates myocardin-induced activation of smooth muscle gene expression. J Biol Chem.

[CR80] Lötzer K (2010). Mouse aorta smooth muscle cells differentiate into lymphoid tissue organizer-like cells on combined tumor necrosis factor receptor-1/lymphotoxin beta-receptor NF-kappaB signaling. Arterioscler Thromb Vasc Biol.

[CR81] Lu Y (2010). Kruppel-like factor 15 regulates smooth muscle response to vascular injury–brief report. Arterioscler Thromb Vasc Biol.

[CR82] Lu Y (2013). Kruppel-like factor 15 is critical for vascular inflammation. J Clin Invest.

[CR83] Luther SA (2002). Differing activities of homeostatic chemokines CCL19, CCL21, and CXCL12 in lymphocyte and dendritic cell recruitment and lymphoid neogenesis. J Immunol.

[CR84] Ma J, Liu J, Li T, Ren J (2022). Hsa_circ_0030042 facilitates the proliferation and migration of vascular smooth muscle cells via the miR-514a-3p/FOXO1 axis. J Endovasc Ther.

[CR85] Majesky MW (2007). Developmental basis of vascular smooth muscle diversity. Arterioscler Thromb Vasc Biol.

[CR86] Markmann A (2003). Expression of transcription factors and matrix genes in response to serum stimulus in vascular smooth muscle cells. Eur J Cell Biol.

[CR87] Martin-Garrido A (2013). Transforming growth factor beta inhibits platelet derived growth factor-induced vascular smooth muscle cell proliferation via Akt-independent, Smad-mediated cyclin D1 downregulation. PLoS ONE.

[CR88] Masuda M (2013). PERK-eIF2alpha-ATF4-CHOP signaling contributes to TNFalpha-induced vascular calcification. J Am Heart Assoc.

[CR89] McConnell BB, Yang VW (2010). Mammalian Kruppel-like factors in health and diseases. Physiol Rev.

[CR90] McCullough KD (2001). Gadd153 sensitizes cells to endoplasmic reticulum stress by down-regulating Bcl2 and perturbing the cellular redox state. Mol Cell Biol.

[CR91] Mebius RE (2003). Organogenesis of lymphoid tissues. Nat Rev Immunol.

[CR92] Myoishi M (2007). Increased endoplasmic reticulum stress in atherosclerotic plaques associated with acute coronary syndrome. Circulation.

[CR93] Nagai R (2005). Significance of the transcription factor KLF5 in cardiovascular remodeling. J Thromb Haemost.

[CR94] Nagao M (2020). Coronary disease-associated gene TCF21 inhibits smooth muscle cell differentiation by blocking the myocardin-serum response factor pathway. Circ Res.

[CR95] Newby AC, Zaltsman AB (2000). Molecular mechanisms in intimal hyperplasia. J Pathol.

[CR96] Nurnberg ST (2015). Coronary artery disease associated transcription factor TCF21 regulates smooth muscle precursor cells that contribute to the fibrous cap. PLoS Genet.

[CR97] Owens GK, Kumar MS, Wamhoff BR (2004). Molecular regulation of vascular smooth muscle cell differentiation in development and disease. Physiol Rev.

[CR98] Pan H (2020). Single-cell genomics reveals a novel cell state during smooth muscle cell phenotypic switching and potential therapeutic targets for atherosclerosis in mouse and human. Circulation.

[CR99] Papavassiliou KA, Papavassiliou AG (2016). Transcription factor drug targets. J Cell Biochem.

[CR100] Pesce M, Scholer HR (2001). Oct-4: gatekeeper in the beginnings of mammalian development. Stem Cells.

[CR101] Poole JC, Cromwell SB, Benditt EP (1971). Behavior of smooth muscle cells and formation of extracellular structures in the reaction of arterial walls to injury. Am J Pathol.

[CR102] Puthalakath H (2007). ER stress triggers apoptosis by activating BH3-only protein Bim. Cell.

[CR103] Rader DJ, Daugherty A (2008). Translating molecular discoveries into new therapies for atherosclerosis. Nature.

[CR104] Raines EW, Ferri N (2005). Thematic review series: the immune system and atherogenesis. Cytokines affecting endothelial and smooth muscle cells in vascular disease. J Lipid Res.

[CR105] Raphel L, Talasila A, Cheung C, Sinha S (2012). Myocardin overexpression is sufficient for promoting the development of a mature smooth muscle cell-like phenotype from human embryonic stem cells. PLoS ONE.

[CR106] Rivard A, Principe N, Andres V (2000). Age-dependent increase in c-fos activity and cyclin A expression in vascular smooth muscle cells. A potential link between aging, smooth muscle cell proliferation and atherosclerosis. Cardiovasc Res.

[CR107] Salmon M (2012). Cooperative binding of KLF4, pELK-1, and HDAC2 to a G/C repressor element in the SM22alpha promoter mediates transcriptional silencing during SMC phenotypic switching in vivo. Circ Res.

[CR108] Shankman LS (2015). KLF4-dependent phenotypic modulation of smooth muscle cells has a key role in atherosclerotic plaque pathogenesis. Nat Med.

[CR109] Shigetoshi S (1999). Keratinocyte-specific ablation of Stat3 exhibits impaired skin remodeling, but does not affect skin morphogenesis. EMBO J.

[CR110] Shin SS (2018). Morin inhibits PDGF-induced proliferation, migration, and invasion of vascular smooth muscle cells via modulating p27KIP1, AKT, and MMP-9 activities. Gen Physiol Biophys.

[CR111] Sikorski K (2011). STAT1-mediated signal integration between IFNγ and LPS leads to increased EC and SMC activation and monocyte adhesion. Am J Physiol Cell Physiol.

[CR112] Sikorski K (2011). STAT1 as a novel therapeutical target in pro-atherogenic signal integration of IFNγ, TLR4 and IL-6 in vascular disease. Cytokine Growth Factor Rev.

[CR113] Sun Y (2012). Smooth muscle cell-specific runx2 deficiency inhibits vascular calcification. Circ Res.

[CR114] Sun SY (2021). Nicotinate-curcumin inhibits AngII-induced vascular smooth muscle cell phenotype switching by upregulating Daxx expression. Cell Adh Migr.

[CR115] Sward K (2016). Emerging roles of the myocardin family of proteins in lipid and glucose metabolism. J Physiol.

[CR116] Tabas I, Ron D (2011). Integrating the mechanisms of apoptosis induced by endoplasmic reticulum stress. Nat Cell Biol.

[CR117] Tabas I, Garcia-Cardena G, Owens GK (2015). Recent insights into the cellular biology of atherosclerosis. J Cell Biol.

[CR118] Talasila A (2013). Myocardin regulates vascular response to injury through miR-24/-29a and platelet-derived growth factor receptor-beta. Arterioscler Thromb Vasc Biol.

[CR119] Tanaka T (2008). Runx2 represses myocardin-mediated differentiation and facilitates osteogenic conversion of vascular smooth muscle cells. Mol Cell Biol.

[CR120] Tang RH (2008). Myocardin inhibits cellular proliferation by inhibiting NF-kappaB(p65)-dependent cell cycle progression. Proc Natl Acad Sci U S A.

[CR121] Torella D (2007). Fludarabine prevents smooth muscle proliferation in vitro and neointimal hyperplasia in vivo through specific inhibition of STAT-1 activation. Am J Physiol Heart Circ Physiol.

[CR122] Trion A, van der Laarse A (2004). Vascular smooth muscle cells and calcification in atherosclerosis. Am Heart J.

[CR123] Tucka J (2014). Akt1 regulates vascular smooth muscle cell apoptosis through FoxO3a and Apaf1 and protects against arterial remodeling and atherosclerosis. Arterioscler Thromb Vasc Biol.

[CR124] Valente AJ (2012). Angiotensin II enhances AT1-Nox1 binding and stimulates arterial smooth muscle cell migration and proliferation through AT1, Nox1, and interleukin-18. Am J Physiol Heart Circ Physiol.

[CR125] Vaquerizas JM, Kummerfeld SK, Teichmann SA, Luscombe NM (2009). A census of human transcription factors: function, expression and evolution. Nat Rev Genet.

[CR126] Vengrenyuk Y (2015). Cholesterol loading reprograms the microRNA-143/145-myocardin axis to convert aortic smooth muscle cells to a dysfunctional macrophage-like phenotype. Arterioscler Thromb Vasc Biol.

[CR127] Wang D (2001). Activation of cardiac gene expression by myocardin, a transcriptional cofactor for serum response factor. Cell.

[CR128] Wang Z, Wang DZ, Pipes GC, Olson EN (2003). Myocardin is a master regulator of smooth muscle gene expression. Proc Natl Acad Sci U S A.

[CR129] Wang C (2015). Apelin induces vascular smooth muscle cells migration via a PI3K/Akt/FoxO3a/MMP-2 pathway. Int J Biochem Cell Biol.

[CR130] Wang J (2019). MicroRNA-92a promotes vascular smooth muscle cell proliferation and migration through the ROCK/MLCK signalling pathway. J Cell Mol Med.

[CR131] Wang Y (2019). Smooth muscle cells contribute the majority of foam cells in ApoE (Apolipoprotein E)-deficient mouse atherosclerosis. Arterioscler Thromb Vasc Biol.

[CR132] Wang J (2021). MicroRNA-520c-3p targeting of RelA/p65 suppresses atherosclerotic plaque formation. Int J Biochem Cell Biol.

[CR133] Wen Y (2022). Di-n-butyl phthalate regulates vascular smooth muscle cells phenotypic switching by MiR-139-5p-MYOCD pathways. Toxicology.

[CR134] Weng G (2021). LINC01123 promotes cell proliferation and migration via regulating miR-1277-5p/KLF5 axis in ox-LDL-induced vascular smooth muscle cells. J Mol Histol.

[CR135] Williams H (2010). MMP-7 mediates cleavage of N-cadherin and promotes smooth muscle cell apoptosis. Cardiovasc Res.

[CR136] Wirka RC (2019). Atheroprotective roles of smooth muscle cell phenotypic modulation and the TCF21 disease gene as revealed by single-cell analysis. Nat Med.

[CR137] Xia XD (2021). Myocardin suppression increases lipid retention and atherosclerosis via downregulation of ABCA1 in vascular smooth muscle cells. Biochim Biophys Acta Mol Cell Biol Lipids.

[CR138] Xie Q (2020). LOXL1-AS1/miR-515-5p/STAT3 positive feedback loop facilitates cell proliferation and migration in atherosclerosis. J Cardiovasc Pharmacol.

[CR139] Xu Z (2015). MiR-135b-5p and MiR-499a-3p promote cell proliferation and migration in atherosclerosis by directly targeting MEF2C. Sci Rep.

[CR140] Xu S, Ni H, Chen H, Dai Q (2019). The interaction between STAT3 and nAChRα1 interferes with nicotine-induced atherosclerosis via Akt/mTOR signaling cascade. Aging (albany NY).

[CR141] Xu J (2020). Paired box 9 regulates VSMC phenotypic transformation, proliferation, and migration via sonic hedgehog. Life Sci.

[CR142] Yang SW (2015). Effects of matrix metalloproteinase 13 on vascular smooth muscle cells migration via Akt-ERK dependent pathway. Tissue Cell.

[CR143] Yang T (2020). A novel role of FKN/CX3CR1 in promoting osteogenic transformation of VSMCs and atherosclerotic calcification. Cell Calcium.

[CR144] Yeh CC (2019). Vanadium derivative exposure promotes functional alterations of VSMCs and consequent atherosclerosis via ROS/p38/NF-κB-mediated IL-6 production. Int J Mol Sci.

[CR145] Yoshida T, Kaestner KH, Owens GK (2008). Conditional deletion of Kruppel-like factor 4 delays downregulation of smooth muscle cell differentiation markers but accelerates neointimal formation following vascular injury. Circ Res.

[CR146] Yoshida T, Yamashita M, Horimai C, Hayashi M (2013). Smooth muscle-selective inhibition of nuclear factor-kappaB attenuates smooth muscle phenotypic switching and neointima formation following vascular injury. J Am Heart Assoc.

[CR147] Yu K, Zheng B, Han M, Wen JK (2011). ATRA activates and PDGF-BB represses the SM22alpha promoter through KLF4 binding to, or dissociating from, its cis-DNA elements. Cardiovasc Res.

[CR148] Yu H (2018). FOXO3a (Forkhead Transcription Factor O Subfamily Member 3a) links vascular smooth muscle cell apoptosis, matrix breakdown, atherosclerosis, and vascular remodeling through a novel pathway involving MMP13 (Matrix Metalloproteinase 13). Arterioscler Thromb Vasc Biol.

[CR149] Zhang LL (2020). CircRNA-PTPRA promoted the progression of atherosclerosis through sponging with miR-636 and upregulating the transcription factor SP1. Eur Rev Med Pharmacol Sci.

[CR150] Zhang QJ (2002). Differential gene expression in vascular smooth muscle cells in primary atherosclerosis and in stent stenosis in humans. Arterioscler Thromb Vasc Biol.

[CR151] Zhang XH (2015). TMEM16A and myocardin form a positive feedback loop that is disrupted by KLF5 during Ang II-induced vascular remodeling. Hypertension.

[CR152] Zhang YN (2016). Phenotypic switching of vascular smooth muscle cells in the ‘normal region’ of aorta from atherosclerosis patients is regulated by miR-145. J Cell Mol Med.

[CR153] Zhang R (2017). MiR-448 promotes vascular smooth muscle cell proliferation and migration in through directly targeting MEF2C. Environ Sci Pollut Res Int.

[CR154] Zhang L (2019). LncRNA LEF1-AS1 regulates the migration and proliferation of vascular smooth muscle cells by targeting miR-544a/PTEN axis. J Cell Biochem.

[CR155] Zhang D (2021). Cyclin G2 promotes the formation of smooth muscle cells derived foam cells in atherosclerosis via PP2A/NF-κB/LOX-1 pathway. Ann Transl Med.

[CR156] Zhao G (2012). Activation of nuclear factor-kappa B accelerates vascular calcification by inhibiting ankylosis protein homolog expression. Kidney Int.

[CR157] Zheng B (2011). HDAC2 phosphorylation-dependent Klf5 deacetylation and RARalpha acetylation induced by RAR agonist switch the transcription regulatory programs of p21 in VSMCs. Cell Res.

[CR158] Zheng B (2018). Regulatory crosstalk between KLF5, miR-29a and Fbw7/CDC4 cooperatively promotes atherosclerotic development. Biochim Biophys Acta Mol Basis Dis.

[CR159] Zhou AX (2015). C/EBP-homologous protein (CHOP) in vascular smooth muscle cells regulates their proliferation in aortic explants and atherosclerotic lesions. Circ Res.

[CR160] Zhu L (2019). Hyperhomocysteinemia induces vascular calcification by activating the transcription factor RUNX2 via Krüppel-like factor 4 up-regulation in mice. J Biol Chem.

